# Microscopic and molecular analysis of *Babesia canis* in archived and diagnostic specimens reveal the impact of anti-parasitic treatment and postmortem changes on pathogen detection

**DOI:** 10.1186/s13071-017-2412-1

**Published:** 2017-10-18

**Authors:** Doroteja Huber, Ana Beck, Željka Anzulović, Daria Jurković, Adam Polkinghorne, Gad Baneth, Relja Beck

**Affiliations:** 1 Department of Veterinary Pathology, Faculty of Veterinary Medicine, University of Zagreb, Vjekoslava Heinzela 55, 10000 Zagreb, Croatia; 2 Faculty of Veterinary Medicine, University of Zagreb, Vjekoslava Heinzela 55, 10000 Zagreb, Croatia; 3 Department for Bacteriology and Parasitology, Croatian Veterinary Institute, Savska cesta 143, 10000 Zagreb, Croatia; 4 Centre for Animal Health Innovation, University of the Sunshine Coast, Sippy Downs, Australia; 5 Koret School of Veterinary Medicine, Hebrew University, Rehovot, Israel

**Keywords:** *Babesia canis*, Merozoite morphology, Postmortem cytological detection, Influence of imidocarb dipropionate, Genotyping

## Abstract

**Background:**

Classification of *Babesia* parasites has traditionally relied on morphological differentiation based on piroplasm size and shape. Molecular typing has subsequently revealed a more complex taxonomy for these piroplasms than previously thought. To evaluate the factors that influence the morphology of *Babesia* species upon microscopic examination and hence, their taxonomic classification, we performed detailed characterizations of piroplasms from archival and prospective collections of cytological samples of dogs with piroplasmosis before and after death. Merozoite morphology and time of parasite disappearance following imidocarb dipropionate was also investigated.

**Methods:**

The study was divided into a (i) review of archived cytological slides from confirmed cases of canine piroplasmosis, and (ii) a prospective study of smears and tissue imprints from 15 recently necropsied dogs. The latter group could be further sub-divided into a non-treated group and an imidocarb dipropionate-treated group. Exact times of treatment before death were reviewed. Additional blood smears prepared from the live dogs and taken before therapy were also evaluated in the latter group. Parasite burden per each slide was determined in both studies. The shape and size of merozoites were described from blood smears taken while the dogs were alive and from different organs during necropsy. The results of all measurements were statistically analyzed.

**Results:**

The morphology and size of merozoites from live dogs corresponded to that of previously described ‘large’ *Babesia*. The morphology and size of merozoites were significantly different (*P* < 0.001) in postmortem samples, however, and more consistent in shape and size with piroplasm cells previously referred to as ‘small’ *Babesia*. PCR and sequencing confirmed *B. canis* as the causative agent of disease in all investigated dogs, including in postmortem negative tissue imprints from dogs treated at least 24 h before death.

**Conclusions:**

Changes in the morphology of ‘large’ *B. canis* to ‘small’-like *Babesia* observed by light microscopy appear to represent a common postmortem change. Classification of *Babesia* parasites into ‘large’ and ‘small’ *Babesia* using only microscopy of postmortem slides should be treated with caution. PCR-based methodologies for detection and molecular typing of *Babesia* spp. may prove valuable for investigating suspected cases of babesiosis following necropsy.

**Electronic supplementary material:**

The online version of this article (10.1186/s13071-017-2412-1) contains supplementary material, which is available to authorized users.

## Background

Piroplasms are globally distributed, obligate intracellular hemotropic parasites of vertebrates from the genera *Babesia*, *Theileria* and *Cytauxozoon* [1, 2]. The name piroplasm comes from the fact that, within erythrocytes, the parasites often appear as pear-shaped on Romanowsky-stained cytological slides following microscopic examination [3]. Microscopic examination has played a central role in the early taxonomic classification of parasites in the genus *Babesia*, with these piroplasms primarily divided into ‘large’ and ‘small’ species based on the shape and size of their intra-erythrocytic stages [4–6] (Additional file 1: Table S1). Further taxonomic classification is not possible without the use of molecular typing approaches [7].

In domesticated dogs, babesiosis is an important and life-threatening hemolytic disease where > 10% of dogs may die despite treatment [8]. In regions endemic for canine babesiosis, every necropsied dog found with jaundice, anaemia, splenomegaly or pigmenturia is suspected to be a potential case of babesiosis, requiring further confirmation of possible protozoal aetiology. For this purpose and on the basis of morphological classification, all ‘large’ forms of *Babesia* in dogs were previously considered to be *Babesia canis* whereas all ‘small’ forms were designated as *Babesia gibsoni* [5]. Subsequently, the use of molecular techniques has enabled further differentiation of ‘large’ *Babesia* into a range of species including *Babesia canis*, *Babesia vogeli*, *Babesia rossi* and *Babesia caballi*, amongst others [9–11]. Within the ‘small’ *Babesia* group, the use of molecular methods has revealed more complexity, including the re-classification of previous ‘small’ *Babesia* species (e.g. *Babesia vulpes*) [12] into new species and, indeed, even different genera (e.g. *Babesia microti*-like’s provisionally classification as *Theileria annae*) [13].

Postmortem identification of *Babesia* organisms is traditionally performed by microscopic examination of tissue imprints, smears and scrapings for morphological classification [14, 15]. Beyond the issues with the accuracy of this classification scheme, the reliance on cytology as an antemortem and postmortem diagnostic tool is problematic in dogs treated during the hemolytic disease with drugs such as imidocarb dipropionate or other antipiroplasmic drugs. These drugs rapidly decrease the numbers of viable merozoites present in the circulation leading to parasite clearance [16] and raise the potential for ‘false-negative’ results following microscopic examination. As an alternative to microscopic examination, molecular detection of pathogens from stained slides or tissue imprints has been shown to be a useful tool for pathogen genotyping for protozoan parasites such as *Leishmania* or *Cryptosporidium* [17–19], particularly for retrospective cases where only cytological slides may be available instead of fresh or frozen tissue [19, 20].

Here, we compared and contrasted the use of microscopic and molecular characterization of piroplasms from stained archival slides and tissues originating from dogs with piroplasmosis. In doing so, the impacts that time of dog’s death and treatment with imidocarb dipropionate had on the microscopic detection and description of these piroplasms were also assessed.

## Methods

### Samples

Samples for analysis in this study were divided into (i) retrospective samples in the form of archival slides and; (ii) prospectively collected samples from recently necropsied dogs. The former group (Group A), consisted of 14 archived Romanowsky-type stained cytological slides, previously microscopically confirmed to be positive for canine piroplasmosis (Table 1). Slides either consisted of clinical blood smears (CBS; *n* = 5) or different postmortem organ imprints (*n* = 9) as described in Table 1.

**Table 1:** Parasite burden and genotyping results from archival cytological slides (Group A)

Samples	Tissue^a^	Year of preparation	Parasite burden	PCR protocols^b^			
				*cytb*	1	2	3
Postmortem samples							
A-1	Kidney	2003	286 (H)	+	+	+	+
A-2	Kidney	2004	527 (VH)	+	–	+	–
A-3	Spleen	2007	134 (M)	+	+	+	+
A-4	Spleen	2008	4 (L)	+	–	+	+
A-5	Spleen	2009	97 (L)	+	–	+	+
A-6	Spleen	2010	32 (L)	+	–	–	+
A-7a	Kidney	2014	43 (L)	+	–	+	–
A-7b	Myocardium	2014	63 (L)	+	+	+	+
A-8	Liver	2014	439 (VH)	+	+	+	+
Clinical samples							
A-9	CBS	2003	160 (M)	+	–	+	–
A-10	CBS	2007	78 (L)	+	+	+	+
A-11	CBS	2010	143 (M)	+	–	–	+
A-12	CBS	2011	112 (M)	+	–	–	+
A-13	CBS	2012	82 (L)	+	–	–	–

^a^Tissue used for cytological slide preparation

^b^Protocol 1: conventional *Babesia/Theileria* PCR using BAB1 and BAB2 primers for amplification of 560 bp 18S rRNA; Protocol 2: conventional *Babesia/Theileria* PCR after DNA concentration and purification using BAB1 and BAB2 primers for amplification of 560 bp 18S rRNA; Protocol 3, nested *Babesia/Theileria* PCR for amplification of 1750 bp 18S rRNA and 560 bp 18S rRNA in the first and second round, respectively

*Abbreviations*: CBS, clinical blood smears made from the cephalic vein blood of clinical patients; L, low parasite burden; M, moderate parasite burden; H, high parasite burden; VH, very high parasite burden; *cytb*, mammalian cytochrome *b*

The prospective study (Group B) included 143 postmortem slides and eight related CBS cytological slides obtained from different tissues of 15 dogs with gross findings consistent with hemolytic disease, necropsied during a one-year period from November 2014 to November 2015 (Table 2). Materials used in this study came from necropsies performed upon owners’ request. All available clinical data regarding treatment and exact time of death were obtained from the referring veterinarians or the dog’s owners (Table 2). Slides in Group B could be further subdivided based on whether the slides were prepared from dogs that received imidocarb dipropionate treatment before death (treated dogs, subgroup T) or were untreated (subgroup NT). In most cases, 10 cytological slides were obtained from each dog of subgroups NT and T, including postmortem blood smears, scrapings from the myocardium and skeletal muscle, and touch imprints from bone marrow, brain, kidney, liver, lungs, lymph node and spleen. For molecular confirmation of piroplasmosis in Group B, both kidney and myocardium tissues were also collected from all carcasses and stored at -20 °C. Fresh tissues were sampled from the exact cut surface location on the organs where the myocardium scrapings and kidney imprints were obtained for the purpose of preparing slides. In eight cases (dogs NT-1, T-1, T-2, T-3, T-5, T-6, T-7 and T-9), a blood sample was also collected in the clinic into an ethylenediaminetetraacetic acid containing tube during the course of the disease or prior the imidocarb dipropionate treatment and stored at -20 °C for molecular analysis. In all eight cases mentioned above, a piroplasmosis positive CBS was available for microscopic reevaluation. Exceptions were dogs T-4 and T-8 for which CPBs were not submitted, although were found positive for the presence of piroplasms by referring clinicians who discarded the slides after microscopic confirmation of diagnosis.

**Table 2:** Parasite burden, treatment data and time of necropsy for the non-treated (NT) and treated (T) subgroups of dogs in Group B

No.	CBS	Postmortem samples										Treatment timing^a^	Time of necropsy^b^
		Blood	Bone marrow	Brain	Kidney	Liver	Lung	Lymph node	Myocardium	Skeletal muscle	Spleen		
NT-1	na	na	27 (L)	492 (VH)	83 (L)	59 (L)	158 (M)	na	342 (VH)	0	41 (L)	na	36
NT-2	na	36 (L)	324 (VH)	481 (VH)	423 (VH)	163 (M)	319 (VH)	114 (M)	361 (VH)	109 (H)	320 (VH)	na	0
NT-3	na	43 (L)	32 (L)	72 (L)	40 (L)	49 (L)	39 (L)	0	57 (L)	0	104 (H)	na	36
NT-4	na	29 (L)	17 (L)	84 (L)	58 (L)	52 (L)	60 (L)	0	52 (L)	na	39 (L)	na	50
NT-5	na	49 (L)	22 (L)	235 (H)	202 (H)	68 (L)	79 (L)	54 (L)	226 (H)	159 (H)	137 (H)	na	48
NT-6	159 (M)	125 (M)	347 (VH)	286 (H)	320 (VH)	157 (H)	82 (L)	24 (L)	na	123 (H)	109 (H)	na	40
T-1	81 (L)	29 (L)	207 (H)	161 (M)	207 (H)	139 (M)	41 (L)	217 (H)	232 (H)	205 (H)	184 (M)	6	17
T-2	138 (M)	25 (L)	322 (VH)	478 (VH)	413 (VH)	193 (M)	90 (L)	56 (L)	361 (VH)	0	309 (H)	6	21
T-3	94 (L)	47 (L)	21 (L)	312 (VH)	92 (L)	83 (L)	118 (M)	34 (L)	134 (M)	48 (L)	57 (L)	10	2
T-4	na	0	0	0	0	0	0	0	0	0	0	10	6
T-5	164 (M)	103 (M)	0	0	0	0	8 (L)	0	0	0	0	24	0
T-6	51 (L)	0	0	0	0	0	0	0	0	0	0	24	11
T-7	47 (L)	0	0	0	0	0	0	0	0	na	0	24	1
T-8	na	0	0	0	0	0	0	0	0	na	0	24	3
T-9	73 (L)	0	0	0	0	0	0	0	0	na	0	48	20

^a^Hours before death

^b^Hours after death. Infection with *B. canis* was confirmed by PCR in organ samples from all dogs

*Abbreviations*: No., dog number; CBS, clinical blood smears made from the cephalic vein blood of clinical patients; na, not applicable; L, low parasite burden; M, moderate parasite burden; H, high parasite burden; VH, very high parasite burden

### Cytological evaluation of clinical and postmortem samples

Regardless of sampling technique (blood smears, tissue imprints or scrapings), cytological slides from all groups were air dried, stained with a Romanowsky-type stain and stored at room temperature in a dry and dark place protected from dust.

Light microscopy evaluation involved detection of piroplasms using an objective magnification of 40× to search the entire surface of the slide. When detected, the morphology of merozoites was described using an oil immersion objective (magnification 100×), and piroplasms were described using terms such as pear-shaped, round, oval, pleomorphic or signet ring-shaped (termed trophozoite in some studies). The dominant morphologic type of piroplasms found was estimated, evaluating up to 100 merozoites present per slide in 10 slides from 10 different tissues from each necropsied dog. The sizes of merozoites expressed in μm were measured with the software Cell^B (Olympus Soft Imaging Solutions GmbH, Muenster, Germany) for up to 100 merozoites on CBS and tissue slides. The location of merozoites (intraerythrocytic or free in the background of the cytologic slide), as well as the maximum number of parasites, present within single erythrocytes, was recorded per each slide, respectively. Parasite burden per each slide was scored as ‘low’ if the total number of counted merozoites found was ≤ 100, ‘moderate’ (101–200 merozoites), ‘high’ (201–300 merozoites) and ‘very high’ (> 300 merozoites). Criteria for estimation of the total number of merozoites were determined by counting of intraerythrocytic and free merozoites visible in high-power fields (magnification 40×) in 4000 erythrocytes. The number of merozoites rather than a number of parasitized erythrocytes was used because of merozoites on postmortem-prepared samples, especially in cases of postmortem autolysis, tend to be free and extracellular rather than in the erythrocyte. A falsely low parasite burden was avoided by counting intraerythrocytic as well as extracellular merozoites. As organ slides contain somatic cells, a variable number of fields were investigated so that 4000 erythrocytes and their immediate surroundings were evaluated per each tissue slide.

### Molecular analysis

DNA was extracted from stained cytological slides from Group A. Before extraction of DNA from cytological slides, 30 μl of ATL buffer (Qiagen, Hilden, Germany) was applied to each slide. Slides were then gently scraped with a sterile, disposable scalpel into sterile 1.5 ml tubes (Eppendorf, Hamburg, Germany). Additionally, 150 μl ATL buffer was added to the tube containing the sample to obtain a total of 180 μl. After adding 20 μl of proteinase K (Qiagen), samples were vortexed for 15 s and pelleted by brief centrifugation. Digestion was performed at 56 °C for 45 min. Extraction of DNA was performed using the QIAamp DNA Mini QIAcube Kit with Blood and body fluids protocol (Qiagen), according to the manufacturer‘s instructions, on an automated system (Qiacube, Qiagen).

In both subgroups of Group B (NT and T), where available, DNA was also extracted from 200 μl of blood collected at the clinic from eight dogs (NT-6, T-1, T-2, T-3, T-5, T-6, T-7 and T-9) using the Blood and body fluids protocol and following the manufacturer’s instructions (Qiagen). Twenty mg of fresh frozen kidney and myocardium tissues collected at necropsy from all dissected dogs were digested for four hours in 180 μl ATL buffer with 20 μl of proteinase K at 56 °C. Extraction of DNA was performed using an automated system and the Tissue protocol with same Kit, according to the manufacturer’s instructions (Qiagen).

The final volume of all DNA extractions was 100 μl. Distilled water was used as a control in each round of DNA extraction. To prevent contamination, all procedures (DNA extraction, PCR preparation, the addition of DNA template to PCR reactions and PCR and capillary electrophoresis) were performed in separate rooms using disposable pipette tips, gloves and aprons. Positive and negative controls (Mastermix with dH_2_O instead of DNA, Mastermix with dH_2_O used as a control in DNA extraction) were included in all amplifications.

Cytological slides from Group A were first subjected to a control PCR for amplification of the mammalian cytochrome b gene, using the primers Cyt b1 (5′-CCA TCC AAC ATC TCA GCA TGA TGA AA-3′) and Cyt b2 (5′-GCC CCT CAG AAT GAT ATT TGT CCT CA-3′), which amplify a fragment of 359 bp of the cytochrome B gene from mammals [21], to assess the potential presence of PCR inhibitors in DNA extracted from archival Romanowsky-type stained slides in Group A. All samples from Group A which were positive for mammalian cytochrome b were further subjected to three different PCR protocols for detection of the presence of *Babesia* DNA. The first protocol (Protocol 1) was a conventional PCR containing 5 μl of extracted DNA from slides for amplification of *Babesia/Theileria* DNA using the forward BAB1 5′-GTC TTG TAA TTG GAA TGA TGG-3′ and the reverse BAB2 5′-CCA AAG ACT TTG ATT TCT CTC-3′ for amplification of 560 bp of the 18S rRNA following the protocol used by Beck et al. [9]. The second protocol (Protocol 2) used an identical conventional PCR protocol (Protocol 1), but it was performed on purified and concentrated DNA (from 100 μl to final volume of 40 μl) with a QIAquick PCR Purification Kit (Qiagen), according to the manufacturer’s instructions. The third PCR protocol (Protocol 3) was a nested PCR. The first reaction in the nested PCR was carried out on 2 μl of DNA with the forward CRYPTO F 5′-AAC CTG GTT GAT CCT GCC AGT AGT CAT-3′ and reverse CRYPTO R 5′-GAA TGA TCC TTC CGC AGG TTC ACC TAC-3′ primers [22] for amplification of 1730 bp of the 18S rRNA gene. In the second nested PCR reaction, 5 μl of PCR product from the first reactions were added and amplified with primers BAB1 and BAB2. For Group B, DNA from tissues and blood from subgroups NT and T were subjected to a conventional PCR reaction for amplification of *Babesia/Theileria* DNA using Protocol 1 described above. A final volume of 50 μl of PCR mixture consisted of 25 μl Go*Taq*® Colorless Master Mix and DNA/RNAase free water (Promega, Madison, WI, USA), 1 μl of 10 pmol of each primer and 5 μl of extracted DNA.

Results of PCRs were evaluated by capillary electrophoresis with QIAxcel (Qiagen) using the QIAxcel DNA Fast Analysis kit, alignment markers (DNA QX Alignment Marker 15 bp/3 kb) and marker sizes (QX DNA Size Marker 50–3000 bp). Amplified samples were purified using EXOSAP-it® (USB® Products AffyInc., Ohio, USA), according to the manufacturer’s instructions, and sequenced in both directions by Macrogen Inc. (Amsterdam, The Netherlands). Sequences were assembled using the SeqMan Pro software, edited with the EditSeq software (Lasergene, DNASTAR, Madison WI, USA) and compared with available sequences using BLAST.

### Statistical analysis

Statistical analyses were carried out using the Statistica 13.2 software (Dell, USA). Range and mean values were determined with the descriptive statistics command and rounded to two digits after the decimal point. Normality of distribution was determined by the Kolmogorov-Smirnov test, and none of the investigated groups followed a normal distribution (*P* < 0.05). The nonparametric Mann-Whitney U-test was used for calculation of statistical significance of the differences in parasite sizes. A *P*-value < 0.05 was considered as statistically significant.

## Results

### Cytological evaluation of clinical and postmortem samples from archival and prospectively collected canine samples

**Fig. 1:**
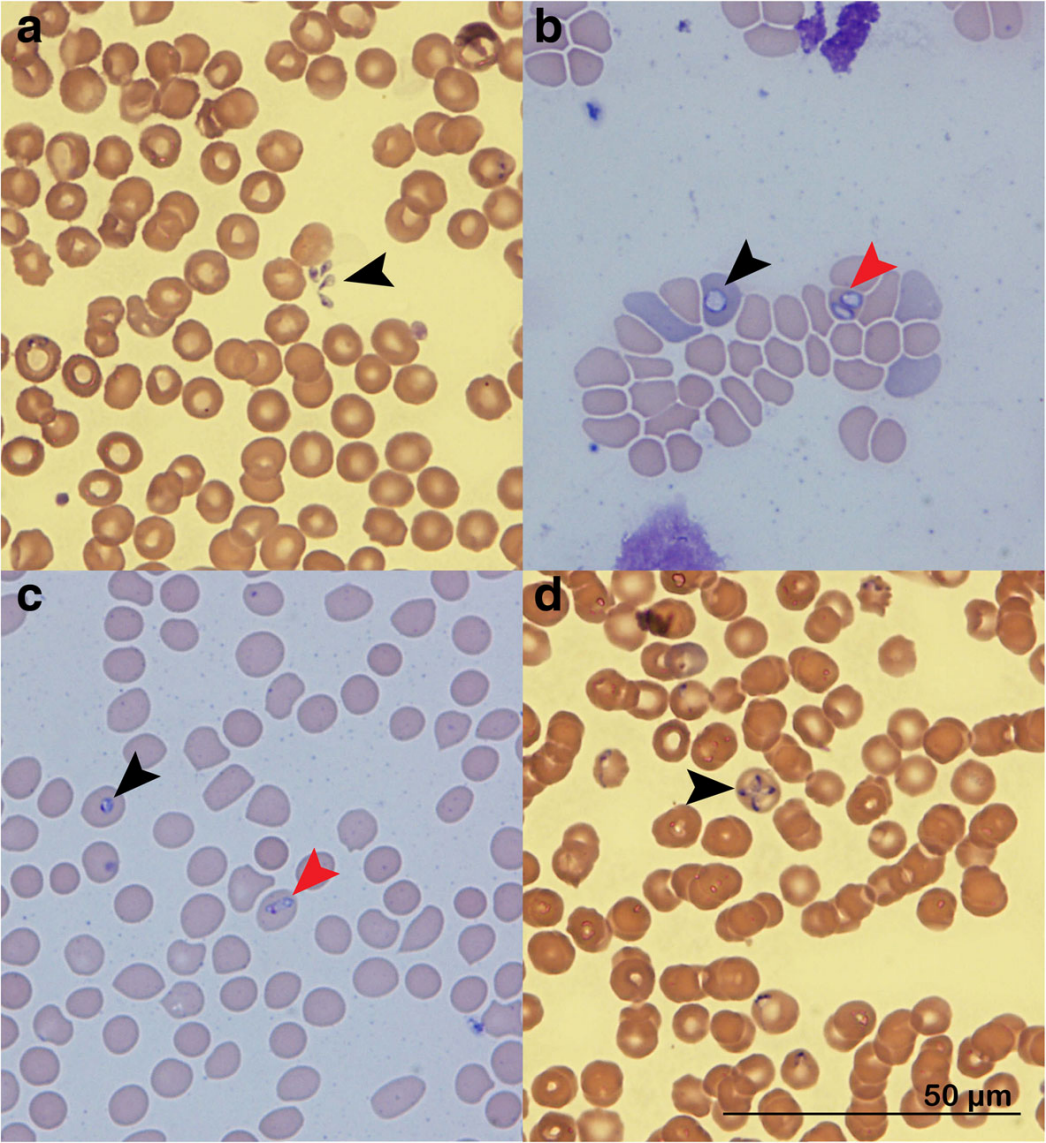
Images of blood smears from dogs before treatment, Romanowsky-type staining, 100×. **a** Four pear-shaped extracellular merozoites (*arrowhead*) measuring 2.66 × 1.45 μm, 2.55 × 1.2 μm, 3.22 × 1.85 μm and 3.15 × 1.21 μm, moderate parasite burden, dog A-9. **b** A reticulocyte containing one round merozoite (*black arrowhead*) measuring 3.42 μm and an erythrocyte containing one oval merozoite measuring 4.03 × 2.05 μm and one round merozoite measuring 2.58 μm (*red arrowhead*), low parasite burden, dog T-1. NCB11 (Nikon’s blue) filter. **c** Two erythrocytes containing one signet-ring trophozoites-like parasite (*black arrowhead*) measuring 1.8 μm and one pleomorphic merozoite (red arrowhead) measuring 2.13 × 1.67 μm, low parasite burden, T-1. NCB11 (Nikon’s blue) filter. **d** An erythrocyte containing four piriform merozoites (*black arrowhead*) measuring 2.53 × 1.2 μm, 1.96 × 1.40 μm, 2.23 × 1.41 μm and 2.21 × 1.15 μm with Maltese cross-like formation, moderate parasite burden, dog A-9

Merozoites detected in CBS slides from archival slides (Group A) and prospectively collected slides (Group B) showed the same range of morphologies. The dominant morphology was pear-shaped (Fig. 1a), but parasites also showed round (Fig. 1b), oval (Fig. 1b), pleomorphic (Fig. 1c) or signet ring-shaped morphologies which could be considered trophozoites (Fig. 1c). Forms resembling the Maltese cross (Fig. 1d) were also observed in a few erythrocytes of some dogs. Merozoites measured 3.75 μm in length (range 1.54–4.2 μm, *n* = 417) by 1.88 μm in width (range 1.2–2.7 μm; *n* = 417) in Group A, and 3.1 μm (range 1.4–5.7 μm, *n* = 800) in length by 2.0 μm in width (range 1.5–2.5 μm; *n* = 800) in both subgroups (NT and T) of Group B. The morphology and size of merozoites are consistent with the previous descriptions of ‘large’ *Babesia* [4] (Additional file 1: Table S1). The merozoites were mostly located in erythrocytes, occasionally in reticulocytes (Fig. 1b) and rarely presented as free, extracellularly (Fig. 1a). Intracellular merozoites were usually paired (Fig. 1b), but they could also be single (Fig. 1c) and rarely four in forms resembling the Maltese cross (Fig. 1d).

**Fig. 2:**
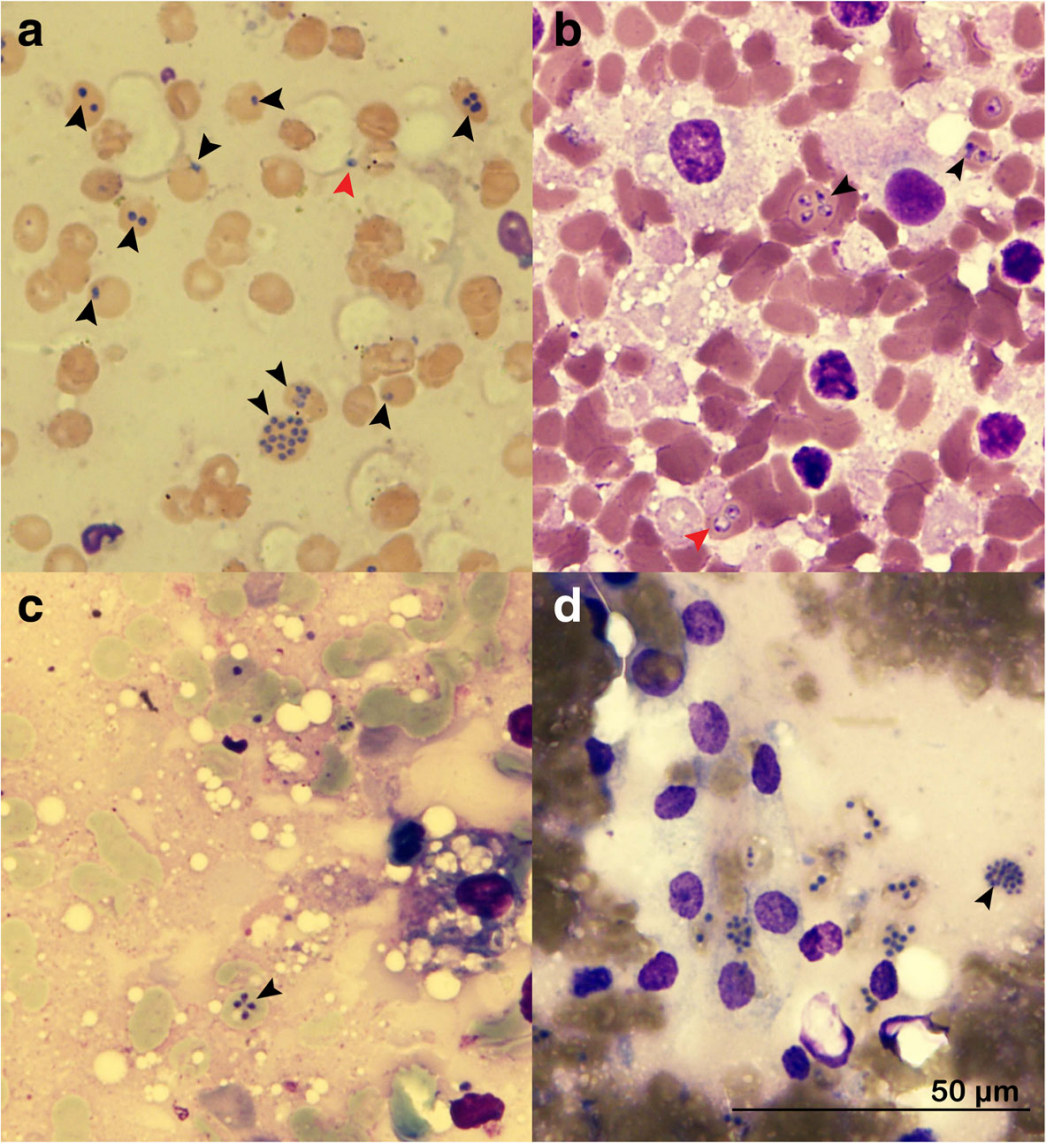
Images of tissue imprints obtained during the necropsy, Romanowsky-type staining, 100×. **a** Nine erythrocytes containing from between one to 16 round *B. canis* merozoites (*black arrowheads*) and one free round merozoite (*red arrowhead*) measuring 0.87–1.2 μm, high parasite burden in kidney tissue imprint, dog A-2. **b** Two erythrocytes (*black arrowhead*) containing two to four pleomorphic merozoites measuring 1.87–2.0 μm in length and 1.0–1.45 μm in width and one erythrocyte (*red arrowhead*) containing two signet-ring shaped merozoites measuring 1.77–1.87 μm, moderate parasite burden in spleen imprint, dog A-3. **c** A Maltese cross-like formation of merozoites measuring 1.57 × 0.96 μm, 1.23 × 0.96 μm, 1.40 × 1.02 μm and 1.02 × 0–96 μm within one erythrocyte (*arrowhead*), moderate parasite burden in liver imprint, dog NT-2. **d** An erythrocyte containing 20 round merozoites (*arrowhead*) measuring 0.5–1.4 μm, high parasite burden in kidney imprint, dog A-1

To compare the potential changes in the morphology of detected piroplasms in postmortem samples, we examined nine archival tissue imprints from Group A (Table 1) and 139 Group B postmortem samples obtained from the six dogs in the NT subgroup (*n* = 56 slides) and nine dogs in the T subgroup (*n* = 83 slides), respectively (Table 2). In these slides, piroplasms were dominantly found as pairs or in more numerous numbers after binary division within the erythrocytes (Fig. 2a, d).

**Fig. 3:**
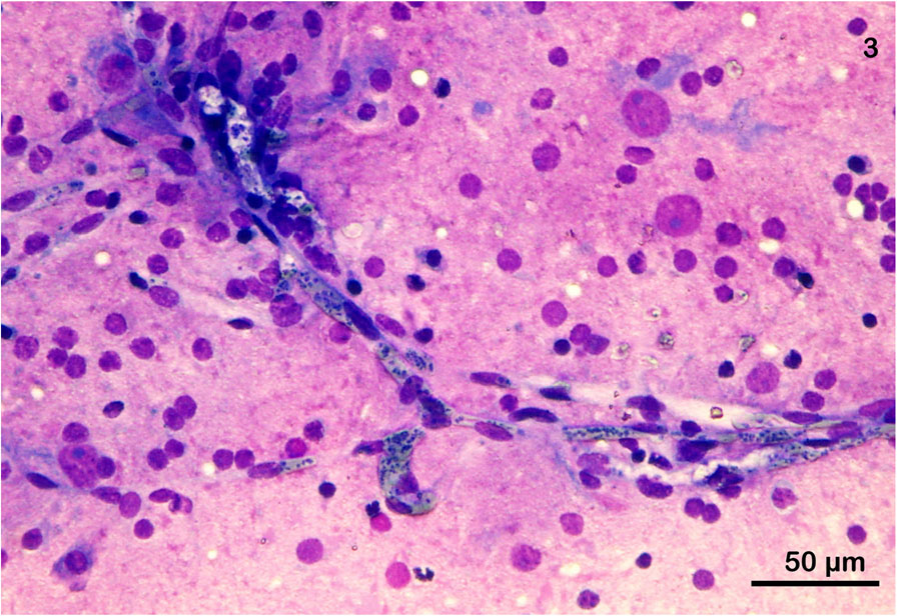
Image of brain imprint obtained postmortal. Magenta background reflecting proteins and lipids from neuropil containing numerous nucleoli of glia cells. Irregular and parallel branching of capillary segments containing numerous erythrocytes heavily parasitized with a round to oval deep blue merozoites, very high parasite burden, dog NT-1, Romanowsky-type staining, 40×

**Fig. 4:**
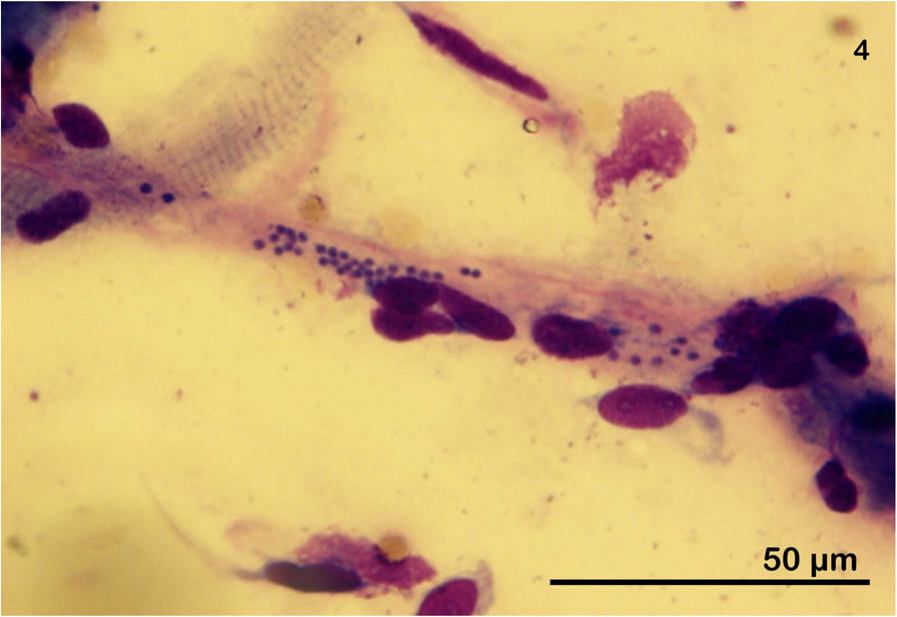
Image of myocardial scraping obtained postmortal. Multiple fibrocytes and a single muscle cell fragment showing transversal striation. A single capillary segment containing free round deep blue merozoites, very high parasite burden, dog NT-1, Romanowsky-type staining, 100×

Merozoites showed consistently different morphologies in all postmortem samples, similarly to that previously described for ‘small’ *Babesia* (Additional file 1: Table S1), when compared to the corresponding CBS slide for sampled dogs. In the postmortem slides, merozoites were shrunken, dominantly round (Figs. 2a, 3 and 4), but also pleomorphic (Fig. 2b), signet ring-shaped (Fig. 2b) or piriform in Maltese cross-like formation (Fig. 2c). In both Group A (3.75 ± 0.53 μm) and Group B (3.1 ± 0.73 μm), the mean length of merozoites in CBS smears was significantly longer (*U*_*A*_ = 0.00, *n*_1A_ = 417, *n*_2A_ = 538, *P*_*A*_ < 0.001; *U*_*B*_ = 4606.5, *n*_1B_ = 800, *n*_2B_ = 900, *P*_*A*_ < 0.001) than the length of merozoites on the corresponding postmortem tissue slides for Group A (1.2 ± 0.15 μm) and Group B (1.2 ± 0.27 μm). Similarly, the width of merozoites from CBS slides was also significantly (*U*_*A*_ = 8305.5, *n*_1A_ = 417, *n*_2A_ = 538, *P* < 0.001; *U*_*B*_ = 9554, *n*_1B_ = 800, *n*_2B_ = 900, *P* < 0.001) wider in both Group A (1.88 ± 0.41 μm) and Group B (2.00 ± 0.2 μm) slides compared to the corresponding postmortem tissue slides from Group A (1.2 ± 0.15 μm) and Group B dogs (1.2 ± 0.27 μm).

In terms of other variables, notably, when the number of intracellular parasites was higher (five or more; Fig. 3), intracellular merozoites of all groups were uniformly smaller in length (0.8 μm; range 0.7–1.1 μm, *n* = 278 and 0.9 μm; range 0.5–1.2 μm, *n* = 300, respectively). In the extracellular space (Fig. 4), merozoites varied from 0.7 to 1.48 μm (mean 1.2 μm; *n* = 183) in Group A, and 0.5 to 2.0 μm (mean 1.2 μm; *n* = 200) for Group B slides, regardless of their number or organ slide.

The morphology of parasites on postmortem slides also did not appear to be related to the time of necropsy after death (Table 2). Parasites were preserved and of similar morphology in slides originating from fresh carcasses, and also in carcasses of dogs necropsied 48 and 50 h after death (NT-4 and NT-5; Table 2).

To investigate the impact of imidocarb dipropionate treatment on the cytological detection of merozoites within tissue slides prepared at post-mortem (Group B), the presence of merozoites between treated (T) and untreated (NT) subgroups were compared (Table 2). In dogs from subgroup NT, merozoites were present in all or almost all slides from all dogs (Table 2). Negative organ slides in the subgroup NT included the lymph node and skeletal muscle (Table 2). In dogs from subgroup T, the presence of merozoites depended on the time of treatment before death. In two dogs treated six hours before death (T-1 and T-2), merozoites were found in CBS and almost all postmortem tissue slides (Table 2). In dog T-3 treated 10 h before death, merozoites were present in CBS and all postmortem slides, while in another dog (T-4) treated 10 h before death, merozoites were not found in postmortem slides. In the remaining dogs treated 24 to 48 h prior to post-mortem (T-5, T-6, T-7, T-8 and T-9), almost no merozoites could be found in post-mortem slides except for a postmortem blood smear and lung touch imprint from dog T-5 necropsied immediately after death (Table 2). No morphological differences could be observed in slides from T and NT subgroups where merozoites could be observed (data not shown).

Parasite burden in either subgroup was variable and inconsistent in different organ slides from the same dog as well as in the same organs originating from different dogs (Table 2). In general, piroplasms were most easily visualized in bone marrow, brain (Fig. 3), kidney, myocardium (Fig. 4) and the spleen based on merozoite counts across from samples from each tissue (Table 2).

### Molecular analysis of clinical and postmortem samples from archival and prospectively collected canine samples

In Group A, mammalian cytochrome B was successfully amplified from all CBS and tissue slides, confirming the absence of any potential PCR inhibitors. *Babesia/Theileria* DNA was successfully amplified with at least one out of three PCR protocols applied in 13 out of 14 slides analyzed (Table 1) from this group. Specifically, the conventional PCR protocol (Protocol 1) was successful in 35.7% (5/14) of the tested samples, while using Protocols 2 and 3, amplification of piroplasm DNA increased to 71.4% (10/14) PCR positivity although differences in the actual positivity of individual samples were different for these latter two protocols. More detailed examination revealed that, for the tissue slides, a higher sensitivity of *Babesia/Theileria* DNA detection was achieved after implementation of DNA purification and concentration (Protocol 2) with PCR positivity of 88.9% (8/9) compared to 77.8% (7/9) for Protocol 3. Conversely for the CBS, Protocol 3 was found to achieve slightly higher PCR positivity (60%; 3/5) compared to Protocol 2 (40%; 2/5). The success of DNA amplification was not correlated to parasite burden per each slide and DNA was amplified from samples with a low, moderate, high and very high number of merozoites, respectively (Table 1). Sequencing revealed that the DNA fragment amplified from all 13 slides was 100% identical with *B. canis* BBC1 isolate (GenBank: FJ209024). DNA sequence quality in Group A archived samples did not appear to be affected by the duration of archiving (Table 1).

All blood, kidney and myocardium samples from Group B were positive for *Babesia/Theileria* DNA, independent of the time of death or treatment (data not shown). All PCR amplified sequences were 100% identical to the BBC1 isolate of *B. canis* (GenBank: FJ209024).

## Discussion

Morphological differentiation of piroplasms in the genus *Babesia* is still routinely used for speciation, particularly in necropsy samples where samples for molecular diagnostics are typically not collected. In the current study, we detected different morphologies of *Babesia* merozoites in cytological slides from clinical and necropsy specimens from the same dogs. Molecular typing of these cases using DNA extracted from the cytological slides themselves, however, revealed that all piroplasms identified belonged to *B. canis*. This phenomenon was previously reported by Demeter et al. [15] in a puppy that died due to piroplasmosis, but the authors suspected the postmortem change in morphology was an exception and not the rule. Here, we present strong evidence that *B. canis* morphology changes from ‘large’ to apparently ‘small’ forms after the death of the host in both treated and non-treated dogs. The most likely explanation for the change in the size of the *B. canis* merozoites under cytological examination is generalized hypoxia, following the death of the dog [15]. The morphological change is not related to the cause of death or time of death as well as the time of treatment because, in fresh carcasses (i.e. dissected right after death), merozoites were already shrunken in all slides.

All dogs with suspicious piroplasmosis or with confirmed ‘large’ *Babesia* merozoites are routinely treated with the antibabesial drug imidocarb dipropionate in Croatia [23]. Imidocarb dipropionate is the preferred treatment because of its rapid therapeutic response and consistent host clearance of babesial parasites [16]. The absence of merozoites in treated dogs with imidocarb dipropionate can pose a challenge in the cytological diagnosis of piroplasmosis, as demonstrated in the current study. Both dogs treated six hours before death had merozoites in slides from most organs (Table 2). However, treatment of two dogs that died 10 h after drug application resulted in different outcomes: complete disappearance of merozoites in one dog, while the other dog still had visible merozoites on postmortem slides (Table 2). Parasite clearance was confirmed in all dogs treated with imidocarb dipropionate 24 h before death where merozoites were not found in postmortem samples examined. Dog T-5 also belonged to this group of dogs who lived an additional 24 h after imidocarb dipropionate application. Dog T-5 was necropsied minutes after death and, despite treatment, it had the exceptional finding of low numbers of merozoites still present on postmortem blood smear and lung imprint. Therefore, we conclude that postmortem cytological confirmation of babesiosis is unreliable in cases of treatment 24 h or more before death. However, our results show that a suspicion that necropsied dogs died of hemolytic disease caused by *B. canis* can still be confirmed by conventional PCR and sequencing on postmortem obtained tissue as evidenced by the PCR detection of *Babesia* DNA in all of these dogs (Table 2).

In cases where fresh or frozen samples are not available for molecular diagnostics, archived material, such as blood or tissue slides can still be useful for PCR as it is routinely done for parasites from the genera *Plasmodium* and *Leishmania* [19, 20, 24]. Despite archiving of up to 12 years, DNA was still amplified and sequenced from the samples in the current study (Table 1). It seems that Romanowsky-type staining conserves DNA from degrading probably due to the fixation with ethanol [19, 20, 24]. The best results were obtained with the samples from Group A Protocol 2 (purified and concentrated DNA) and Protocol 3 (nested PCR) likely due to higher amounts of available DNA in the sample and a higher number of amplification cycles performed, enhancing the sensitivity of the reactions. Protocol 2 was more suitable for amplification of DNA from tissue slides, while the Protocol 3 (nested) PCR, although equally successful as protocol 2 overall, was better in amplifying DNA from blood smears.

## Conclusions

In the current study, we have confirmed that a change in morphology of ‘large’ *B. canis* to ‘small’-like *Babesia* morphology, as observed by light microscopy, consistently takes place postmortem. These findings also suggest that classification of *Babesia* parasites into ‘large’ and ‘small’ *Babesia* could lead to false conclusions on species involved if postmortem slides are analyzed unless genotyping is performed. We, therefore, recommend that in necropsy cases with a history of antibabesial treatment or without historical data, PCR diagnostic testing should be utilized over microscopy since, after 24 h, it was almost impossible to detect merozoites in tissue slides.

## Additional file

Additional file 1: Table S1. Literature review of *Babesia* classification into ‘large’ and ‘small’ based on size of merozoites. (XLSX 12 kb)

## Abbreviations

CBS: clinical blood smears from the cephalic vein of clinical-ill dogs
*cytb*: mammalian cytochrome *b*
na: not applicable
